# A Superior Wisdom Tooth Discharged from the Nasal Passages; with Remarks

**Published:** 1887-01

**Authors:** John S. Marshall

**Affiliations:** Chicago, Ill.


ARTICLE VI.

### A SUPERIOR WISDOM TOOTH DISCHARGED FROM THE NASAL PASSAGES; WITH REMARKS.

BY JOHN S. MARSHALL, M. D., CHICAGO, ILL.

[Read in the Section on Dental and Oral Surgery at the Thirty-Seventh Annual Meeting of the American Medical Association.]

I am indebted to Dr. Emma F. Gaston, of Chicago, a member of this Association, for the history of the following very remarkable case, and which I present with a few remarks in explanation of the peculiar symptoms, the probable course taken by the tooth, and which finally resulted in its being discharged from the posterior nares.

"Mrs. B., aged 62, in October, 1884, suffered intensely from what she supposed to be 'a severe cold in the head.'

The pain in the right orbital region was at times excruciating, but not more severe than she had often experienced in other portions of the face and right ear, during the previous ten years. Attacks of facial neuralgia and otalgia had been so frequent during these years, that she had become quite disheartened and had no expectation of ever being freed from them.

"Medical aid had only temporarily alleviated her suffering, and she had become 'tired of consulting physicians' about 'neuralgia.' In this attack the usual domestic remedies for a cold, and an anodyne application for neuralgia had been resorted to without alleviation of symptoms. The right nostril became slightly swollen and completely obstructed. Breathing was almost impossible when the lips were closed.

"After a few days of discomfort, the patient made an unusually great effort to relieve the nostrils, by alternately coughing, and blowing the nose. Suddenly she felt something fall upon her tongue. Spitting the mass into the wash-bowl, she heard a clicking sound, which caused her to examine it. Imagine her surprise at finding a large right superior wisdom tooth covered with fœtid pus. The relief of the facial pain was immediate, 'the cold in the head' was explained, and probably much of the 'torture' she had endured periodically for years, was also accounted for.

"This very unexpected experience led the patient to recall an incident which occurred about twenty-six years before. In the year 1854, thirty years previous to the expulsion of this 'eccentric tooth,' she had all her upper teeth removed. Wore a 'temporary set' of artificial teeth for several months, then had a 'permanent set,' on gold plate, which she wore with great comfort, for four years. Then a tumefaction appeared upon the right superior maxilla, near the tuberosity; and she jested about 'cutting a wisdom tooth.' Consulted her dentist about the 'plate.' He found that in perfect condition, and assured her there was no prospect about a tooth, and was unable to assign any satisfactory cause for the suffering.

"After a few days the annoying swelling and tenderness disappeared, and from that time until 1884, no thought was ever given to those unexplained symptoms.

"In May, 1884, the patient suffered from an abscess in the right ear. After the abscess ruptured an unusual amount of hæmorrhage from the ear occurred. The patient became very much weakened by it, and a physician remained at her bedside for hours.

"Previous to this date she had gradually lost the power of hearing on that side, and was afterwards totally deaf in that ear. There had also been a slight fœtid discharge from the nasal passages for several years, but which was supposed to be the result of a simple catarrhal affection."

*Remarks:* It would seem from the foregoing history that all the pain and discomfort in the right facial region endured by this lady for so many years, was the result of this erratic tooth, and the explanation I would offer is as follows:

The swelling and tumefaction mentioned as having appeared at the posterior portion of the right superior maxilla about five years after the extraction of the superior teeth, viz., twenty-five years ago, was undoubtedly caused by the eruption of this tooth, (and it must have been formed in an inverted position) which, taking a direction upwards and forwards, finally pierced the floor of the antrum of Highmore. The disappearance of the tumefaction, and all unpleasant symptoms speedily passing away, would I think, be a fair inference that this was what occurred. The tooth must, however, have remained in this position for several years, as I think the condition of the tooth would strongly indicate, for you will notice the crown is covered with a thick dark-brown deposit which is very rough upon its surface, while upon its roots there is considerably less, and they have the appearance of being exposed to the effects of the antral secretions for a much shorter period of time. The aural abscess, and this tooth, I think have a very close relationship as regards cause and effect; and I am of the opinion that the abscess in the ear was the result of an abscess at the roots of this tooth, and which discharged its contents into the meatus, and at the same time freed the tooth from its crypt in the maxillary bone and left it loose in the antrum.

The tooth being loose finally found its way to the anterior portion of this cavity and lodged there, and by contact with its nasal wall produced ulceration of this and the inferior turbinated bone, and thus found its way into the nasal passages and into the mouth.

The slight catarrhal (?) discharge which had been persistent for so many years was without doubt the result of the presence of the tooth in the antrum. The record of the case is valuable from the fact that it explains an obstinate case of persistent neuralgia of the trifacial nerve, and indicates the probable cause of a severe aural abscess. The symptoms of the case were, however, so obscure and indefinite in their character as to give no indications of their real cause, and the presence of a tooth or other foreign body in the antrum was not even suspected.—*Jour. Amer. Med. Association.*

